# Acquisition, quality control, and architecture of a large image dataset as a tool for in silico cell biological research

**DOI:** 10.1016/j.mtbio.2025.102352

**Published:** 2025-09-24

**Authors:** Nikita Konshin, Marta Garcia Valverde, Danila Solodennikov, Koen Minartz, Vlado Menkovski, Rosalinde Masereeuw, Shantanu Singh, Silvia M. Mihăilă, Jan de Boer

**Affiliations:** a Department of Biomedical Engineering and Institute for Complex Molecular Systems, Eindhoven University of Technology, Eindhoven, the Netherlands; b Division of Pharmacology, Utrecht Institute for Pharmaceutical Sciences, Utrecht University, Utrecht, the Netherlands; c Department of Mathematics and Computer Science, Eindhoven University of Technology, Eindhoven, the Netherlands; d Imaging Platform, Broad Institute of Harvard and MIT, Cambridge, MA, United States of America

**Keywords:** TopoChip, Podocyte, Morphological fingerprinting, High-content imaging, FAIR data, Quality control, Dataset, Micro-topography, Machine learning

## Abstract

We present a large-scale, standardized image dataset and analysis pipeline designed to enable in silico discovery of cell–material interactions. This resource paper introduces an open, FAIR-aligned framework for acquiring, curating, and analyzing high-content imaging data of kidney podocytes cultured on 2176 micro-topographical surfaces using the TopoChip platform. Our workflow includes automated imaging, tilt correction, object segmentation, and multi-tiered quality control, resulting in over 5500 morphological features for >1.2 million cells. Structured metadata, standardized file architectures, and ontological annotations ensure that the dataset is fully interoperable and ready for reuse. To illustrate its versatility, we provide examples of how this resource supports machine learning model development, reproducible benchmarking, and hypothesis testing in cell biology and biomaterials science. This dataset and accompanying tools are designed as a foundational reference for the community, enabling scalable, quantitative, and reproducible exploration of how microenvironments shape cell behavior.

## Graphical abstract

**Figure:**
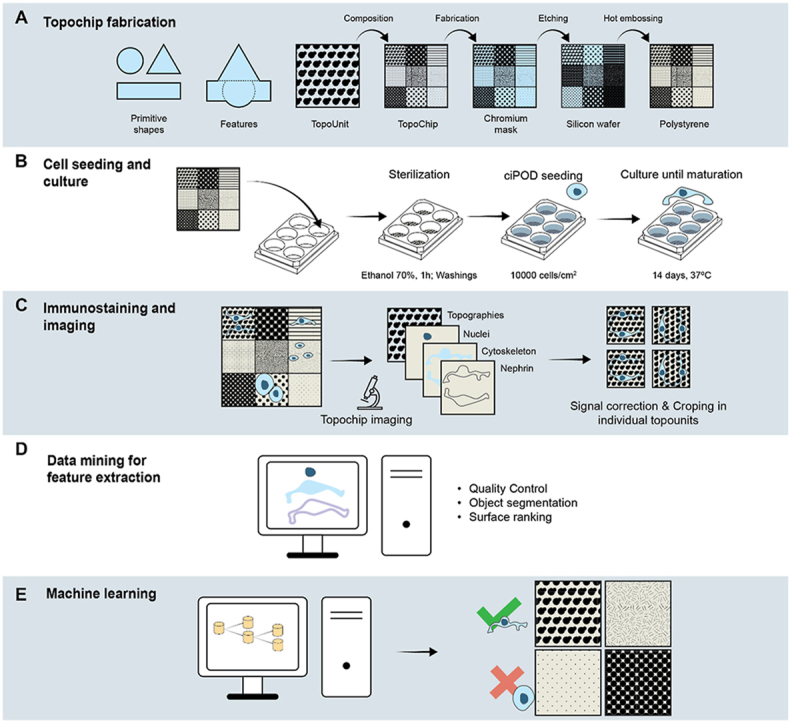

## 1 Introduction

Understanding how cells respond to biomaterials is central to advancing medical implants, regenerative therapies, and tissue engineering. Surface properties of biomaterials—such as topography, chemistry and stiffness—can direct cell behavior in ways that impact adhesion, proliferation, differentiation, and tissue integration. Numerous studies have established correlations between surface characteristics and phenotypic outcomes [[1], [2], [3], [4], [5], [6], [7], [8], [9], [10], [11], [12], [13], [14], [15], [16]] and have yielded important improved biomaterials which have entered the clinic, such as bone-inducing microporous calcium phosphate ceramics, acid-etched titanium dental implants and polymers that prevent biofilm formation [3,6,7,17].

High-throughput screening (HTS) platforms have emerged as powerful tools for probing the relationship between biomaterial surface design and cellular responses and for systematic exploration of biomaterial design space. These platforms present thousands of unique biomaterials, i.e. microfabricated surface topographies or polymers, on a single substrate, enabling parallel analysis of cellular behavior at an unprecedented scale. New materials have been discovered with unique bio-active properties, such as those improving the clonogenic potential of embryonic stem cells [14,16], tuning of the immune system [7,18,19] in vitro and in vivo, and osteoblast and bone physiology [3,7].

The next phase in the development of HTS libraries of biomaterials involves the adoption of standardized, scalable, and reproducible pipelines for data acquisition and analysis. Inspiration for such efforts can be drawn from fields like transcriptomics and drug screening, which have successfully adopted FAIR (Findable, Accessible, Interoperable, and Reusable) data practices, leading to robust repositories and computational frameworks [20,21], which can be reused when new questions arise. For example, transcriptomics leverages curated databases like GEO [22] and Expression Atlas [23] alongside resources such as the Connectivity Map [24] (CMap), which provides a comprehensive set of tools to analyze various biological assays and are widely used by researchers to test and generate new hypotheses. In the field of high content imaging, good examples include the Cell Painting Gallery [25,26] and the Allen Cell Collection [27] which follow FAIR data principles to support hypothesis generation, benchmarking, and tool development. Biomaterial science would benefit from a comparable infrastructure.

A fundamental challenge in biomaterials HTS lies in the complexity of the material surface itself. Unlike flat, uniform well plates made of industry-standard polystyrene or glass, biomaterial libraries are typically manufactured in-house, and feature complex micro- and nanoscale geometries or droplets of polymers, which can introduce artifacts in imaging and complicate downstream analysis. While issues such as autofluorescence, shadowing, variations in z-height, and edge effects can also affect drug screening, they are often exacerbated by the complex nature of biomaterial surfaces, necessitating bespoke imaging and image processing solutions [2,17,18,28]. Furthermore, the spatially heterogeneous nature of these substrates poses challenges for assay uniformity and experimental replication [29].

To date, most data from biomaterial libraries is acquired by high-content imaging (HCI) of fluorescently labeled cells grown on the materials, which require techniques for automated image analysis and feature extraction [2,30]. These methods, inspired by cell profiling approaches such as Cell Painting, allow the capture of rich morphological information across multiple channels and magnifications [30,31]. However, applying these techniques to biomaterials screening requires careful calibration and control, including segmentation protocols that account for substrate-induced distortions and QC filters that can detect imaging artifacts [32,33].

Several studies have mined biomaterial-induced phenotypic datasets using statistical and machine learning (ML) approaches, for instance by building models on protein absorption and cell responses, fouling of bacteria based on mass spectrometry data of polymer surfaces, or our own models on topographical surface design properties and their relation to stem cell physiology [2,29,34,35]. Although these examples give proof of concept, they remain siloed, with limited generalizability due to differences in experimental design, lack of annotated metadata, and absence of public repositories. In contrast to the omics field, where standards in data structure, metadata annotation, and community-driven curation facilitate broader use, biomaterials research lacks such systemic conventions [36,37]. We believe that sharing of best practices, based on detailed experimental description, will assist in reaching consensus on standardization.

Therefore, we describe a protocolized, image-based screening workflow, using cells cultured on the TopoChip platform as an example system to demonstrate these principles. Podocytes, the specialized epithelial cells of the kidney glomerulus, are highly sensitive to microenvironmental cues and exhibit complex morphologies that are readily influenced by substrate properties [2,[38], [39], [40]]. They represent a biologically relevant and morphologically rich model for studying cell–material interactions. By coupling podocyte culture with a diverse set of surface topographies, we generated a large and heterogeneous dataset suitable for downstream phenotypic and mechanistic analysis [18,41].

Beyond demonstrating a specific application, our aim is to contribute a reproducible and extensible workflow that can be adapted to other cell types, imaging modalities, and material platforms. This includes aligning with FAIR data principles and promoting reuse by the broader research community. While our workflow incorporates structures that facilitate data harmonization and metadata organization, we recognize that true interoperability necessitates integration with community-agreed standards, vocabularies, and ontologies. We advocate for the establishment of reference datasets, benchmark challenges, and shared image analysis pipelines to further accelerate discovery in the biomaterials domain [42].

Ultimately, this work highlights the potential of imaging-based, high-throughput phenotyping combined with rigorous data practices to systematically decode cell–material interactions. By enabling scalable, standardized, and quantitative analysis, we move closer to the design of biomaterials that actively instruct cell behaviour. Rather than claiming to fully enable interoperability, we view this work as a step toward future standards-based integration, contributing foundational elements for in silico biomaterials development by bridging biology, materials science, and computational modelling in a unified framework [43].

## 2 Materials and methods

### 2.1 Production and quality control of TopoChips

A detailed description of the surface fabrication procedures was published previously [2], and the corresponding Standard Operating Procedures are available in our lab's GitHub repository (https://github.com/cbite/TopoChip-analysis) and permanently archived on Zenodo (https://doi.org/10.5281/zenodo.17015757). In short, the inverse pattern of the topographies was etched into a silicon wafer by directional reactive ion etching. To facilitate demolding, the wafer was coated with a layer of trichloro(1H,1H,2H,2H-perfluorooctyl)silane (Sigma-Aldrich). Polydimethylsiloxane (PDMS; Down Corning) was cured on the silicon wafer to generate a positive mold and was subsequently used as a template to create a second negative mold in Ormostamp polymer (micro resist technology Gmbh), which serves as the mold for hot embossing of polystyrene (PS) films (Goodfellow). The hot embossing procedure was carried out at 140 °C for 5 min, and with a pressure of 10 Bar and demolding temperature of 90 °C. Before cell culture, the PS topographies and the flat PS surfaces were treated with oxygen plasma for 30 s at 0.8 mBar, 50 sccm O_2_ and 100 W to improve cell adhesion. Quality of the fabricated imprints was assessed using a Keyence VK-H1XM-131 profilometer.

### 2.2 Cell seeding on TopoChips

To stabilize the TopoChip on the bottom of the cell culture well, a cylindrical holder (34 mm outer diameter, 13 mm high) with an open central square (23 mm sides) was designed in SolidWorks and 3D printed in polylactic acid (Ultimaker S3, Ultimaker, Utrecht, NL). Prior to cell seeding, TopoChips and holders were sterilized for 60 min in 70 % v/v ethanol and rinsed three times for 5 min with Hanks' Balanced Salt Solution (HBSS, Cat. No. 14065056; Fisher Scientific, USA). Conditionally immortalized podocytes (ciPODs) were kindly donated by Dr. Moin Saleem from Bristol University with all relevant ethical approval in place [44]. ciPODs will be further be referred to as podocytes. Cells of passage 20 were seeded at a density of 10,000 cells/cm^2^, accounting only for the effective surface in the central opening of the holder. The cells were cultured in RPMI 1640 medium supplemented with 10 % foetal calf serum (FCS), 1 % penicillin/streptomycin (P/S), and 1 % ITS supplement (insulin, transferrin, selenium, Sigma-Aldrich).

After seeding, the plates were gently swayed clockwise and counterclockwise to promote homogeneous distribution of cells on the TopoChip. Cells were maintained at 33 °C in 5 % CO_2_ for two days to allow attachment. They were then transferred to 37 °C for 14 days to induce maturation. Under these conditions, mature podocytes cease proliferation due to inactivation of the temperature-sensitive SV40T antigen and begin to express characteristic podocyte markers [44]. Medium was refreshed bi-weekly. SOPs on cell seeding can be find at our lab's Github: https://github.com/cbite/TopoChip-analysis/blob/main/SOPs/2-bioAssays.7z.

### 2.3 TopoChip fixation and immunostaining

Cells were washed with phosphate buffered saline (PBS, Sigma-Aldrich) 3 times to remove any debris before fixation. Cells were fixed in 4 % paraformaldehyde (PFA) solution (Pierce™ 16 % formaldehyde (w/v), methanol-free, ThermoFisher, MA, USA; diluted with PBS) for 10 min. Next, samples were permeabilized in 0.3 % (v/v) Triton X-100 (Sigma-Aldrich) in Hank's Balanced Salt Solution (HBSS) for 10 min and blocked (blocking buffer containing 2 % w/v bovine serum albumin (Sigma-Aldrich) and Tween-20 (0.1 %, v/v, Sigma-Aldrich) in HBSS) for 30 min. Next, samples were incubated with human anti-nephrin antibody (1:100, AF4269, R&D Systems, Minnesota, USA) diluted in blocking buffer for 60 min at room temperature. After washing 3 times for 5 min with PBS-Tween (0.1 % v/v), donkey anti-sheep AF 594 (1:200, A11016, ThermoFisher, MA, USA) and phalloidin-iFluor 647 (1:1000, ab176759, Abcam, Cambridge, UK) were added and samples were incubated for 60 min at room temperature. Finally, samples were incubated with 4′,6′-diamidino-2-phenylindole (DAPI) diluted 1:1000in blocking buffer (D1306, Invitrogen, MA, USA) for 8 min. Stained TopoChips were mounted on glass slides using Prolong gold antifade medium (Cell Signaling Technology, MA, USA). Samples were stored at 4 °C, protected from light and imaged between 30 and 100 days post-staining.

### 2.4 Image acquisition

A Nikon Ti2 inverted high-throughput screening microscope was utilized for both brightfield and fluorescence imaging to visualize and quantify nephrin, F-actin, and DNA in fixed and stained samples. For each chip, a preliminary scan was performed at 10 × magnification to assess the overall staining quality and detect staining artifacts, which we defined as irregularities such as uneven staining intensity, non-specific binding of the dyes, or areas of fluorescence that were inconsistent with the expected homogeneous signal over the whole chip. This assessment was performed both in the DAPI and phalloidin channels to ensure consistent nuclear and cytoskeletal staining. If no significant artifacts were detected and staining quality was deemed sufficient—defined as uniform staining across the sample with minimal background fluorescence—imaging was then switched to 20 × magnification. Imaging in 20 × magnification was conducted using LIVE mode, which provides real-time acquisition with optimized exposure settings to minimize photobleaching and maintain high-resolution output. Acquisition settings were adjusted to ensure that overexposed pixels were minimized (<1 % of the total image pixels) across all 10 chips, preserving accurate intensity quantification and avoiding signal saturation. The Nikon Ti2 imaging plugin *Highlight Overexposed Pixels* was used to dynamically control exposure parameters. Overexposure thresholding is critical to prevent the loss of detail in high-intensity regions, ensuring reliable downstream image analysis and consistent data quality across all scanned chips. Based on visual sharpness of the phalloidin signal, a tilt heatmap—a visual representation of variations in focal plane height across the chip—was generated for each chip. This was achieved by using corner TopoUnits as reference points to define the autofocus range. The purpose of generating the tilt heatmap was to identify and correct unevenness in the imaging plane caused by minor variations in TopoChip surface thickness or mounting. Tilt correction ensured consistent focus across the entire imaging area, and improved the reliability and accuracy of the acquired images.

After setting up the autofocus, the Nikon *Large image acquisition* module was used for automated imaging. The protocol included the following steps.

• Z-Stack Parameters: The autofocus channel was defined to ensure optimal focus during imaging. A Z-stack range of 16 μm was specified, with a step size of 4 μm. This meant that the microscope captured images at multiple focal planes within this range at intervals of 4 μm. This approach enabled the capture of three-dimensional details of the sample and accounted for variations in sample thickness, ensuring that all structures of interest were in focus.
• Channel Selection: The excitation and emission spectra for each channel were as follows: nephrin: Excitation 540–568 nm/Emission 579–640 nm; DAPI: Excitation 379–405 nm/Emission 420 nm; phalloidin: Excitation 590–645 nm/Emission 680 nm.

Corresponding filters were used to capture these wavelengths during imaging, ensuring high specificity and minimal overlap between channels.

• Area Definition: Regions of interest for imaging were defined manually based on the layout of the chip and the expected areas of cell growth. These regions were selected to ensure complete cover age of the chip while excluding empty or irrelevant areas.
• Binning: Binning refers to the process of combining adjacent pixels during image acquisition to increase sensitivity and reduce file size. We used 1 × 1 binning to preserve the maximum spatial resolution, which was essential for accurately segmenting fine podocyte features.
• Stitching: The large image acquisition module automatically combined individual images into seamless, larger reconstructions. An overlap of 10 % between adjacent images was specified to ensure accurate alignment and to minimize artifacts at the boundaries of stitched regions.
• File Management: File naming conventions were standardized to enable clear identification of the chip, imaging region, and channel. The file name format used was: Marta_NK_[chipX]_zstackDNA, Mito,Nephrin,ER.nd2

Upon starting acquisition, files were initially saved locally in.nd2 format. These files were then transferred to a local server for temporary storage. Subsequently, raw data backups were archived for long-term cold storage. This workflow ensured secure data management and accessibility for downstream analysis. Detailed protocol: https://github.com/cbite/TopoChip-analysis/blob/main/SOPs/3-imaging.7z.

### 2.5 Image quality control and preprocessing

All acquired images underwent a systematic quality control process to ensure consistency and reliability for downstream analysis. Initially, the large TopoChip stitched images were visually inspected for regions with abnormally high staining intensity, which could indicate inhomogeneous cell seeding or staining artifacts. No such areas were observed in the dataset. The correct orientation of TopoChips was verified by ensuring that the TopoUnit containing the flat surface was localized in the lower-right corner of the image. Large TopoChip images were aligned to an XY grid using ImageJ for accurate spatial referencing. To capture the full nephrin signal across all focal planes, z-stacks of nephrin images were superimposed and saved as z-projections. Maximum intensity projection was applied using ImageJ's max projection function, which selects the highest intensity pixel at each location. This method was chosen to emphasize regions with strong nephrin staining, typically observed in podocyte foot processes. Next, images were cropped in ImageJ to capture the TopoChip area, excluding any peripheral regions. From the resulting full TopoChip images, individual TopoUnit images were extracted using a MATLAB script, topocropper.m. The file name format used was: [Channel]_[ChipID]_Col[ColumnNumber]_Row[RowNumber]_Seq[SequenceNumber]_[Stain].tif. This process generated a dataset comprising 30 TopoChips (10 chips × 3 stains), resulting in 66 rows × 66 columns × 10 TopoChips × 3 stains = 130,680 images. A detailed description of the cropping process, along with the MATLAB script and guidelines, is available on GitHub (https://github.com/cbite/TopoChip-analysis/blob/main/scripts/TopoCropper.m).

### 2.6 Object segmentation and feature extraction

Object segmentation and feature extraction were performed to quantify morphological and intensity-based features from the cells grown on the TopoChip. A CellProfiler pipeline was developed and optimized to enable consistent and robust segmentation of cellular structures. The pipeline is available on our lab's GitHub repository (https://github.com/cbite/TopoChip-analysis/blob/main/scripts/ciPODs_podocyte_segmentation_for_TopoChip_screens_NK_16_01_2024.cpproj). The segmentation workflow included the following steps: illumination correction to mitigate background intensity variations, and object segmentation using DAPI as the primary stain (nucleus), with phalloidin and nephrin serving as two distinct secondary stains (cell body and protein expression, respectively). Parameter optimization for object detection, such as size thresholds and intensity cutoff values, was performed using CellProfiler's “Test mode”, based on visual inspection of representative images.

To ensure biological relevance and avoid boundary artifacts—segmentation errors caused by cells or nuclei that are partially visible at the edges of an image or TopoUnit, we implemented exclusion rules for improperly segmented objects. Specifically, in the *IdentifyPrimaryObjects* module, only nuclei between 15 and 65 pixels in diameter were retained, and all objects touching the image border were discarded. Likewise, in the *IdentifySecondaryObjects* module (run separately for phalloidin and nephrin), secondary objects in contact with the walls separating the TopoUnits were excluded. If a secondary object was discarded for this reason, the corresponding primary object was also removed. This two-tier filter ensured that only fully contained and well-defined cells were retained for analysis.

Following segmentation optimization on a manually curated subset of 50 images, the final pipeline was applied to the full dataset. More than 1000 TopoUnits were randomly sampled and visually evaluated by two independent observers (NK and MGV) to validate segmentation fidelity. Agreement on segmentation accuracy was >95 %; occasional discrepancies were resolved through consensus discussion, ensuring consistency. The processed images yielded over 1.2 million segmented cells and more than 1500 features per cell across three channels. Feature data were exported as.csv files for each chip and later merged into a comprehensive database for downstream analysis. The complete dataset supporting this study has been deposited in Zenodo as.h5 file [45].

### 2.7 Data curation and quality assessment of cell image features

Following feature extraction, a data curation step was performed to ensure the quality and consistency of the extracted cell image features. A Python script (https://github.com/cbite/TopoChip-analysis/blob/main/scripts/removeOutliers.py) previously developed for TopoChip screens was used to curate segmentation results. This process involved removing artifacts and ensuring that only high-quality segmentations were retained for analysis. A set of metrics was chosen to identify and exclude artifacts, including Cell Count (to remove over- and underpopulated TopoUnits), Focus Score (to exclude out-of-focus cells) and Nucleus-to-Cell Body Area Ratio (Identifying objects with abnormal ratios). Next, Interquartile Range (IQR) thresholds were applied to define acceptable ranges for cell count and focus score. We tested multiple cut off values and visually inspected flagged outliers to ensure that only biologically implausible data were removed while retaining normal variation. Of the 39,204 images (for each stain) originally processed, TopoUnits that fell outside these IQR boundaries were discarded, resulting in a final dataset of 36,024 images (for each stain). All visualization and outlier annotation steps were conducted in Python (Matplotlib, Seaborn). The final curated dataset is stored in two formats: a CSV file (3.13 GB) and an HDF5 file (1.45 GB) for flexible downstream access [46]. Detailed protocols: https://github.com/cbite/TopoChip-analysis/blob/main/SOPs/4-analysis.7z, SOP_4_1 to SOP_4_4 (Standard Operating Procedure).

### 2.8 Statistical analysis for reproducibility

To assess the reproducibility of cellular responses across replicate surfaces, we applied a two-step filtering approach based on statistical and signal-based criteria. First, the Anderson-Darling (AD) test was used to compare the distribution of replicate measurements for each surface design (TopoUnit). This non-parametric test evaluates whether replicates — typically duplicated TopoUnits positioned across the chip — follow the same statistical distribution. A low p-value (p < 0.05) indicates inconsistency between replicates, possibly due to experimental artifacts or biological variability, and those surfaces were excluded. Second, we calculated the Signal-to-Noise Ratio (SNR) for each surface to quantify the stability of the cellular response. While the AD test focuses on distributional agreement, SNR measures the magnitude of variability relative to the mean, providing a complementary filter. A low SNR suggests high noise and low reproducibility even if distributions are statistically similar. Together, these metrics ensure that retained surfaces exhibit both consistent distribution and stable signal across replicates. In total, 343 surfaces were removed based on cell count, 148 based on nuclear area, and 385 based on nuclear form factor (Table 1). This dual-filtering strategy, applied across all surfaces regardless of chip position, enhances the robustness of our dataset for downstream analysis. Detailed protocols: https://github.com/cbite/TopoChip-analysis/blob/main/SOPs/4-analysis.7z, SOP_4_5.

**Table 1:** Number of removed surfaces.

Hyperparameter	AD p-value (0.05; 1)	SNR (2; 20)
Cell count	296	47
Nuclear area	143	5
Nuclear form factor	101	284

### 2.9 Machine learning approach and model training

We trained a LightGBM classifier to predict binarized biological readouts (e.g. cell morphology or cell count) from topography design descriptors (TDDs) [47]. Input data consisted of TDDs as features, and the binarized readouts of cell morphology as the target variable. The top and bottom N performing surfaces were selected based on various cellular characteristics, such as cell count, to generate these binarized readouts. Features were standardized using StandardScaler (z-score normalization) to ensure uniform scaling. For data splitting, the dataset was first divided into training and testing sets with a test size of 20 %, using stratified sampling based on the target variable (Y) to maintain class proportions. Subsequently, the initial test set was further split into a new test set and a validation set, with 20 % of this subset allocated for validation, also employing stratified sampling based on its target variable (y_test). This resulted in an approximate distribution of 80 % for training, 16 % for testing, and 4 % for validation. A LightGBM classifier was trained to classify these top and bottom-performing surfaces. Hyperparameter tuning was performed using Optuna's Tree-structured Parzen Estimator (TPE) sampler, optimizing for balanced accuracy and ROC-AUC. Key hyperparameters tuned (Table 2) included boosting type (GBDT, DART, RF), number of leaves (2–2500), maximum depth (−10 to 900), learning rate (1e-12 to 0.99), and number of estimators (100–24,100). Optuna's HyperbandPruner was used to terminate underperforming trials early, thereby efficiently accelerating the search for optimal hyperparameters. Performance was assessed using balanced accuracy, precision, recall, and ROC-AUC. Cross-validation was applied to ensure robust performance estimates. Feature importance was extracted from LightGBM's built-in metric, and SHAP values were computed to interpret the contribution of individual descriptors. AUC-ROC curves were plotted to compare training and test performance. The top 10 most predictive descriptors were identified for biological interpretation. Analysis was conducted in Python using the LightGBM, Optuna, scikit-learn, and SHAP libraries. All computations were performed on high-performance computing infrastructure.

**Table 2:** Hyperparameters of the LightGBM model general settings.

Hyperparameter	Value
Boosting Type	gbdt
Objective	binary
Number of Leaves	191
Max Depth	118
Learning Rate	0.01679910379082482
Number of Estimators	12042
Random State	42
Data Sample Strategy	goss
Top Rate	0.17018468861347003

Regularization	
Hyperparameter	Value

Lambda L1	14.825534539789798
Lambda L2	95.37157659523345
Linear Lambda	70.86772503160189
Drop Rate	0.73844801734339
Max Drop	1267
Skip Drop	0.2862573476293778
Uniform Drop	False

Tree and Sampling Settings	
Hyperparameter	Value

Deterministic	True
Force Column-wise	True
Min Sum Hessian in Leaf	87.74013625036233
Feature Fraction	0.25455318721130743
Other Rate	0.09885757333923684
Min Data per Group	435
Max Categorical Threshold	1734
Cat L2	85.84302114069115
Feature Fraction by Node	0.375789890524636
Extra Trees	False
Max Bin	387

Computational Settings	
Hyperparameter	Value

Number of Jobs	−1
Cat Smooth	62.80892158399032
Top K	887
CEG Boosting Tradeoff	98.43099413468349
Scale Positive Weight	4.6257547692635566
Sigmoid	64.16478915867698
Verbose	−1

The complete workflow is available on GitHub (https://github.com/cbite/ciPODs_9_PS_TopoChips_SCREEN_NK_MGV_14022025). Detailed protocols: https://github.com/cbite/TopoChip-analysis/blob/main/SOPs/4-analysis.7z, SOP_4_6.

## 3 Results

### 3.1 TopoChip fabrication

**Fig. 1:**
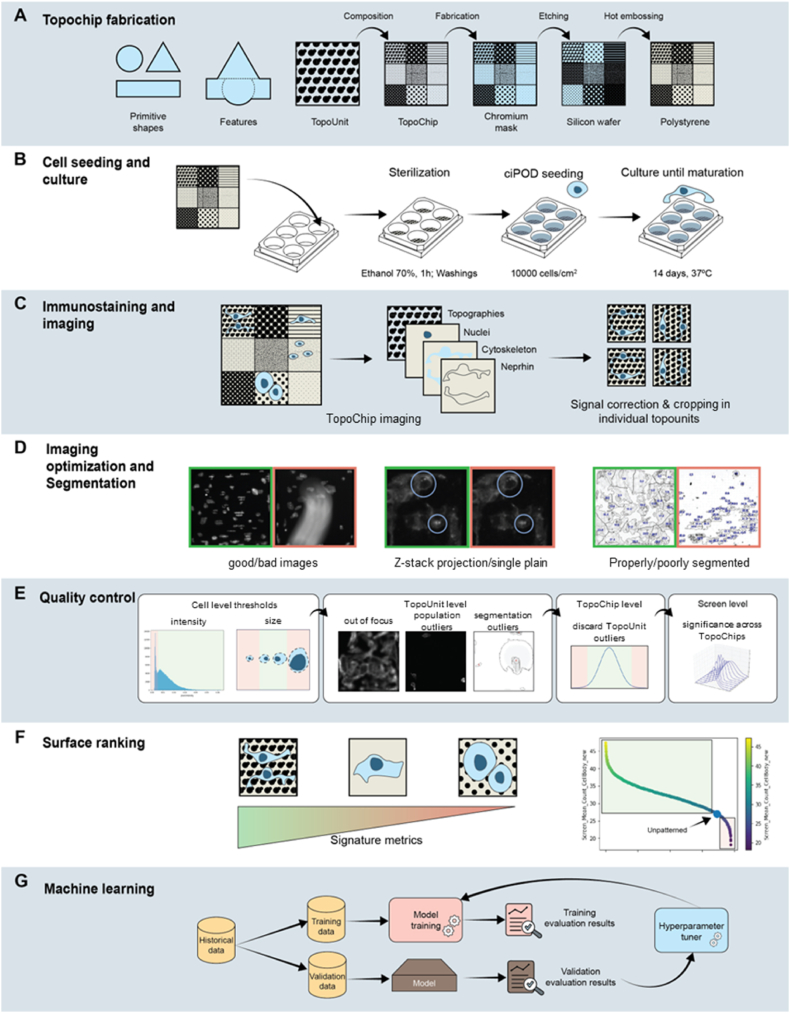
Design, manufacturing and screening of the TopoChip platform.

**Fig. 2:**
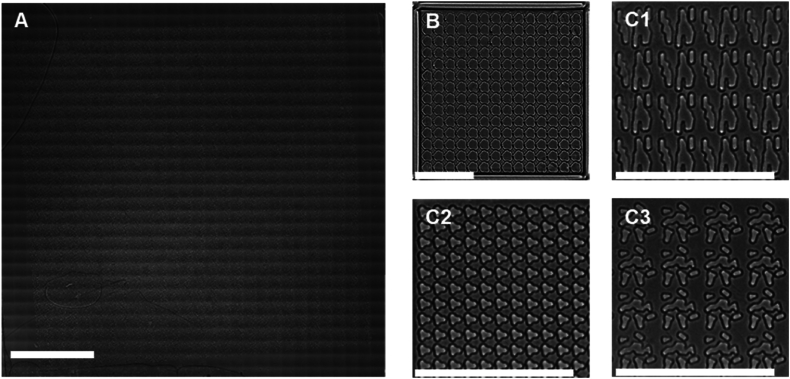
**Brightfield images of TopoChips.** (**A**) Full stitched brightfield image of a polystyrene TopoChip, showing the array of micro-topographies across the chip: 66 rows, 66 columns, comprising 2176 TopoUnits with patterns in duplicate and 4 TopoUnits with a flat surface. The image was generated by stitching multiple smaller images acquired with a 20 × objective, resulting in visible tiling artifacts due to slight variations in illumination and alignment between individual image tiles. (**B**) Single TopoUnit including vertical walls that prevent cellular migration to neighbouring TopoUnits. (**C1, C2, C3**) Selected regions of different TopoUnits, highlighting the individual topographical features. Scale bar represents 0.5 cm (A) and 100 μm (B, C1, C2, C3).

To produce a high quality image data set of podocytes grown on a library of topographies, polystyrene TopoChips with 2176 unique micro-topographies were fabricated and characterized. The workflow (Fig. 1) encompassed topographical pattern design, cell seeding, immunostaining, imaging, quality control, and machine learning. Brightfield imaging (Fig. 2A) confirmed the successful generation of TopoChips, while at higher resolution (Fig. 2B) the precise and reproducible arrangement of individual pillar architecture across the chip was confirmed.

### 3.2 Podocyte morphological changes induced by topography

**Fig. 3:**
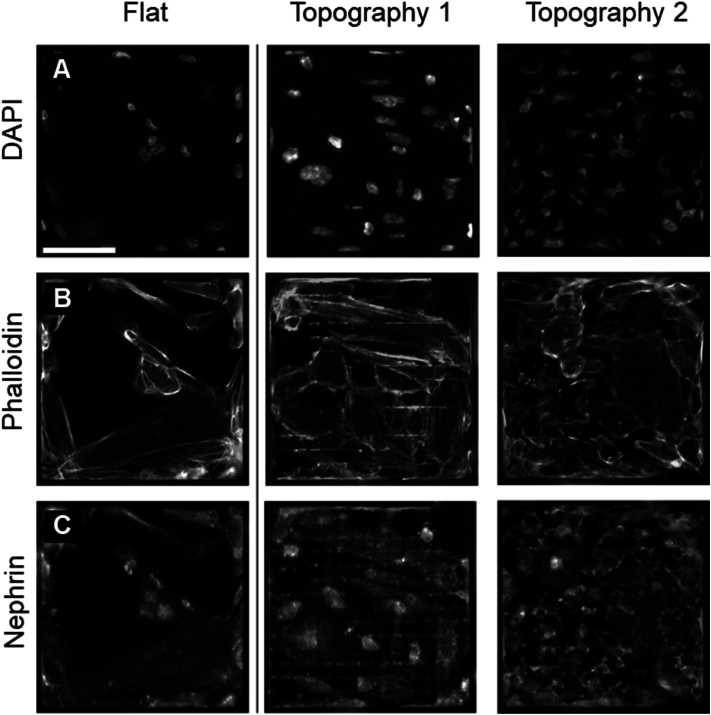
**Podocytes cultured on flat and patterned surfaces.** (**A**) Nuclear DNA staining shows cell distribution and density. (**B**) F-actin staining highlights the organization of the actin cytoskeleton. (**C**) Nephrin illustrates podocyte-specific marker expression. Cells on flat surfaces displayed a uniform pattern, while topographical surfaces induce distinct phenotypic changes in cytoskeletal arrangement, nuclear shape and nephrin expression.

**Fig. 4:**
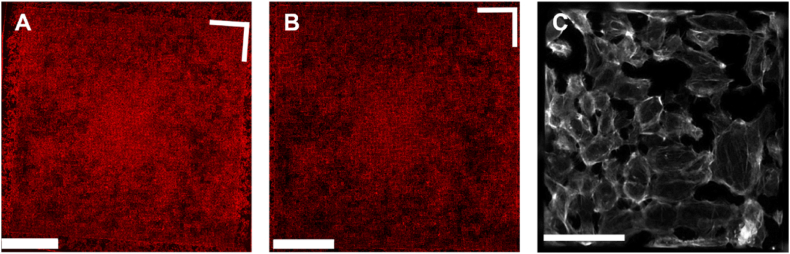
**Processing and multiscale visualization of raw microscopy data. (A)** Unprocessed TopoChip microscopy image (phalloidin staining), depicting the complete array of topographical patterns. A white **L-shaped angle marker** in the top-right corner serves as an **orientation indicator**. **(B)** Cropped and rotated TopoChip image, adjusted for orientation. **(C)** Magnified and processed microscopy image of an individual TopoUnit (phalloidin staining), highlighting detailed actin cytoskeletal organization on a topography. Scale bars represent 0.5 cm (A, B) and 100 μm (C), respectively.

Podocytes were grown on TopoChips for 10 days, fixed and stained for DNA (DAPI), F-actin cytoskeleton (phalloidin), and the podocyte marker nephrin, to investigate whether the response of podocytes to topographical cues can be captured by staining and imaging (Fig. 3). On flat control surfaces, podocytes were typically large cells with an ellipsoid shape and round nuclei. The actin cytoskeleton was organized in stress fibres, sometimes with a clear cortical location. Nephrin signal was detected throughout the cell body at low intensity, with some bright speckles throughout the cell with notable distance relative to the periphery of the cell. On the other hand, we noticed two different classes of podocytes grown on topographies: those where podocytes attached to the top of the pillars and those where podocytes attached to the surface between the pillars. Overall, podocytes on topographical surfaces displayed very irregular nuclei, the area of which was smaller relative to cells grown on flat surfaces. As we noticed before in mesenchymal stem cells, DAPI signal in deformed nuclei was stronger than in nuclei with a normal shape [48], indicating that the topography has an effect on the epigenetic status of the DNA. The cell body of podocytes that attached to the top of the pillars appeared to have a smaller cell area compared to podocytes grown on a flat surface. Also they had stress fibres and we did notice cortical actin staining. When podocytes grew between the pillars, the pillars were clearly visible in the outline of the nuclei and cell bodies. F-actin was organized in clear stress fibres throughout the cell including the cortex. Moreover, some confined podocytes displayed high intensity cortical nephrin signal with small speckles evenly distributed throughout the cell (Fig. 3, topography 1). From these visual observations we conclude that the topographical library induces a heterogenic response in podocyte cell and nuclear shape and nephrin expression pattern (see Fig. 4).

### 3.3 Multiscale analysis and signal localization

**Fig. 5:**
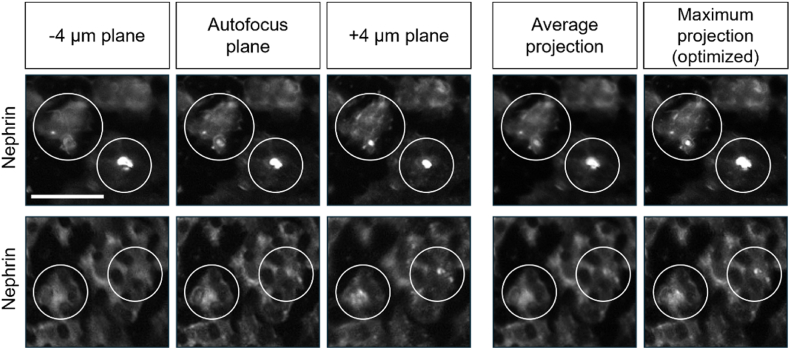
**Nephrin signal acquisition via z-stack projection.** Nephrin immunostaining on two representative TopoUnits (top and bottom rows) at three Z-stack positions (−4 μm, autofocus (0 μm), and +4 μm) and their average and maximum intensity projections. Circles denote regions exhibiting pronounced variations in nephrin signal intensity or localization. Scale bar: 50 μm.

The next phase in generating a library of quantitative data was to acquire the fluorescent signals of the cells and generate high quality images. To this end, 10 polystyrene TopoChips were seeded with podocytes and images were acquired after 10 days of culture. Phalloidin had a high signal-to-noise ratio and good structural contrast, and was thus used to perform autofocus. To capture the volumetric distribution of nephrin at the cell membrane—a feature of high biological relevance—we performed z-stack imaging with 4 μm step intervals. Specifically, we acquired nephrin staining at three distinct z-planes (−4 μm, 0 μm, +4 μm), which revealed clear differences in signal intensity and localization: the −4 μm plane exhibited the lowest overall brightness, the 0 μm plane (determined as the optimal focal plane via a combination of Nikon autofocus and its Focus Surface function) displayed small, round high-intensity specks, and the +4 μm plane was the brightest, revealing even smaller granules. To simplify downstream image processing, we decided to collate the Z stack into one image. To determine whether to use the average or maximum intensity values per pixels, we compared the effect of the ImageJ z-stack projection functions on nephrin signal. We concluded that average projection offers a good general overview, but maximum intensity projection (Fig. 5) retained even the smallest features, and was therefore chosen for downstream analyses. Moreover, the regions of interest (ROIs) highlighted in these panels revealed distinct variations in nephrin expression across the topographical patterns, offering valuable insights into how micro-topographical cues modulate podocyte morphology. Individual images for every stain were stitched to form a whole TopoChip image (Fig. 3A). This outlined the entire array of podocytes on topographical patterns. The white right-angle indicator shows the border of the TopoChip and demonstrates the need for correction of chip orientation and cropping of the image to the size of the TopoChip (Fig. 3B). From each processed whole TopoChip image, we cropped for each of the three channels a total of 4356 images of single TopoUnits (Fig. 3C). Using these setting, we generated a collection of 130,680 images of 2176 different TopoUnits with podocytes, stained with three different dyes.

### 3.4 Optimization of imaging and segmentation pipelines

**Fig. 6:**
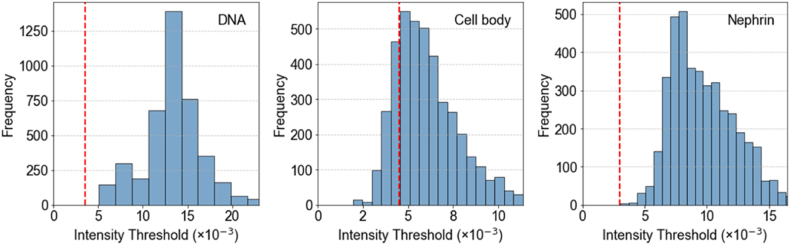
**Intensity threshold distributions for subsampled data.** Thresholds were suggested by the Image Quality Check module, with the final values (red dashed lines) chosen based on visual assessment in CellProfiler's Test mode for optimal segmentation. (For interpretation of the references to color in this figure legend, the reader is referred to the Web version of this article.)

**Fig. 7:**
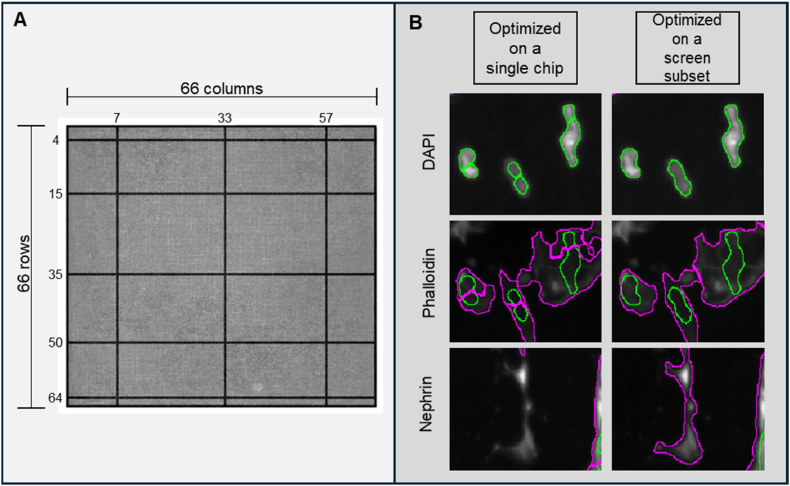
**Pipeline optimization for intensity-based object segmentation.** (**A**) Graphical representation of the grid which was used to select images to set the image analysis pipeline. The grid illustrates the layout and sampling framework applied across all TopoChips to standardize the pipeline across the screen. The numbered slices represent rows and columns included in the optimization dataset. (**B**) Comparison of segmentation pipelines optimized on different training sets. The left column displays results from a pipeline where segmentation thresholds were optimized using images from a **single chip only**, while the right column shows results from a pipeline trained on a **subset of images sampled across the grid in panel (A)**, representing all 10 chips in the screen. Three staining channels are shown: DAPI (top), phalloidin (middle), and nephrin (bottom). **Green outlines** represent segmentation of **DAPI-stained nuclei**, while **magenta outlines** represent segmented **phalloidin** (F-actin) and **nephrin** signals. For transparency and future benchmarking, we also provide a manually annotated subset of 108 TopoUnit images with expert ground-truth segmentations, available via GitHub (https://github.com/cbite/TopoChip-analysis/releases/tag/v1.1.1). (For interpretation of the references to color in this figure legend, the reader is referred to the Web version of this article.)

The next step in our workflow was to define podocytes as unique objects in the images and to extract quantitative features describing fluorescent signals on the 2176 different topographies. In order to standardize data analysis, we developed and optimized an intensity‐based segmentation pipeline using CellProfiler and applied it to every image. To set parameters for segmentation, we created a representative sample pool (RSP) by selecting five rows and three columns from each TopoChip and extracting corresponding images from each chip (Fig. 7A), including all TopoUnits with a flat surface. This RSP totaled 514 images per chip, which is approximately 10 % of all images from a single TopoChip and was used to sample images for optimization of the pipeline. We incorporated a Correct Illumination Function (Regular/Divide) combined with a median filter (manual smoothing filter size = 200) to smooth images and reduce noise. Next, we employed the “Measure Image Quality” module to assess suggested threshold ranges for improved object segmentation across all images, with particular emphasis on nuclear, F-actin, and nephrin signals. We used the pipeline on images from the RSP and obtained suggested intensity thresholds (Fig. 5). Then, we visually inspected primary and secondary object segmentations based on the suggested intensity thresholds and defined threshold values for optimal segmentation. After reviewing the range of suggested thresholds and performing a series of pipeline adjustments, we visually inspected the segmentation quality in CellProfiler's Test mode and manually set the final intensity threshold values, indicated by a red dashed vertical line in the intensity distribution plot (Fig. 6). It is worth mentioning that an adaptive thresholding strategy with window size 400 was used for secondary object segmentation for both nephrin and phalloidin. Fig. 7B compares segmentation results using different threshold settings for nuclei, phalloidin, and nephrin stains; white circles highlight regions where segmentation was enhanced, and the final examples (Fig. 7B) demonstrate accurate segmentation of nuclei (top), phalloidin (middle), and nephrin (bottom) stains using the optimized pipeline. With this CellProfiler pipeline, we processed all 130,680 images, using the DAPI signal to identify primary objects, and both phalloidin and nephrin signals to identify secondary objects. Due to software-related issues, we were not able to retrieve data from one of the 10 TopoChips. In total, we identified 1,213,838 cells. Per cell, CellProfiler extracted 5490 features, resulting in a database of 1,213,838 × 5490 = 6,663,970,620 features. In addition, we obtained information for each of the 130,680 TopoUnits in our library.

### 3.5 Outlier detection and removal

**Fig. 8:**
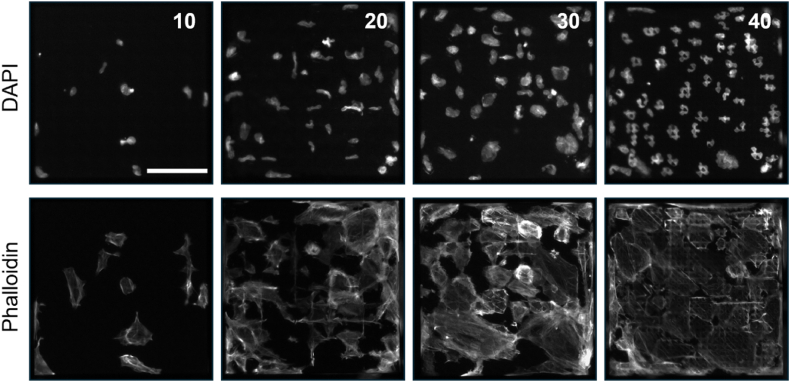
**Variation in cell number across different TopoUnits.** Images illustrate increasing cell densities from left **(10)** to right **(40)**, as indicated by DAPI staining in the top panels. Corresponding actin cytoskeletal organization is shown in the bottom panels (phalloidin staining). Numbers in the upper-right corner represent cell counts per TopoUnit. Scale bar: 100 μm.

**Fig. 9:**
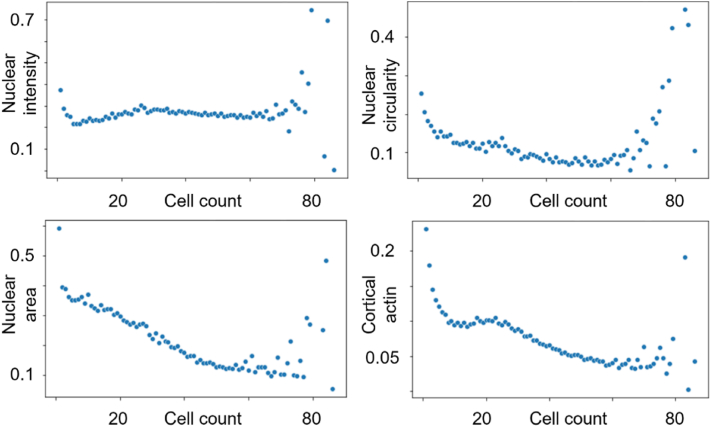
**Coefficient of variation of cell features at different cell counts.** Each dot represents the coefficient of variation (cv), calculated as the standard deviation divided by the mean, for cell morphological metrics measured from approximately 70–80 surfaces (TopoUnits) that share the same cell count. Four morphological features (nuclear intensity, nuclear circularity, nuclear area, and cortical actin) are shown in separate panels. Cell counts represent the average number of cells per surface. The cv is dimensionless and indicates relative variability within each group of surfaces.

To ensure data quality and to remove artifacts, we implemented a multi-tiered quality control (QC) strategy, with detailed procedures described in the Materials and Methods section. This involved QC steps at three levels: TopoChip, object, and TopoUnit. At the TopoChip level, visual inspection confirmed the success of cell culture experiments and stainings across all full TopoChip images, resulting in no exclusions from the dataset. At the object level, automated processing within the CellProfiler pipeline applied stringent morphological criteria. This included defining a specific range for the major axis length of primary objects (nuclei), as well as removing any primary and secondary objects that overlapped with image borders. This approach ensured that only complete and well-segmented nuclei and cell outlines were included, thereby reducing edge effects and artificial cuts. This process identified 1,213,838 primary objects on 9 chips, with visual inspection confirming efficient segmentation. Finally, at the TopoUnit level, cell count served as a crucial QC metric (Fig. 8). TopoUnits with very low or very high cell counts were identified for removal, as these extremes can introduce significant variability or compromise the interpretability of results. Specifically, TopoUnits with very few cells can lead to outliers strongly influencing mean values, while those with very high cell numbers may exhibit multilayering, altering the cell-material interaction environment (Fig. 8). To establish the appropriate range for this metric, we analyzed the coefficient of variance of key CellProfiler features (cortical actin, nuclear form factor, mean DAPI intensity, and cell area) against cell count (Fig. 9). Based on this analysis, all images of TopoUnits with fewer than 11 cells and more than 64 cells were removed, resulting in the exclusion of 5278 images and 63,789 unique objects.

**Fig. 10:**
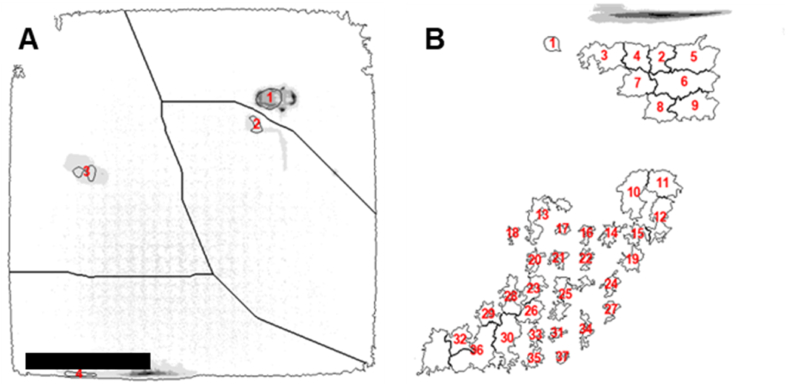
**Examples of mis segmentations removed by the nuclear to cell area ratio**. (**A**) Low nuclear-to-cellular area ratio; (**B**) high nuclear-to-cellular area ratio. Red annotations highlight objects identified as outliers. Scale bar: 100 μm. (For interpretation of the references to color in this figure legend, the reader is referred to the Web version of this article.)

An additional TopoUnit-level metric that proved particularly useful was the nuclear-to-cell area ratio (NtoC), defined as the ratio of the nuclear area to that of the surrounding cytoplasm. This metric was instrumental in identifying segmentation artifacts—instances where the delineated regions did not correspond to biologically plausible cells, which are typically characterized by a nucleus enveloped by a substantial cytoplasmic area. An NtoC value approaching 0 indicates that the nuclear area is significantly smaller than the cytoplasmic area, whereas a value of 1 suggests that the nucleus and cytoplasm occupy approximately equal areas. Such cases often reflect incorrect segmentations where CellProfiler detects primary and secondary objects that did not correspond to actual nuclei or cells. To exclude such biologically irrelevant TopoUnits, we applied a filtering threshold based on the 2.5th and 97.5th percentiles of the NtoC distribution. Using percentile-based thresholds allowed us to adapt the filtering to the observed distribution in each dataset; while this choice was guided by visual inspection rather than a formal optimization, it nevertheless provided stable and reproducible outcomes across experiments. This step effectively removed 2006 images, corresponding to 27,804 unique objects (Fig. 10A and B). TopoUnit QC removed 7284 images with 91,593 unique objects, and resulted in a curated database of 31,920 images with 1,122,245 cells.

### 3.6 Structure and richness of the dataset

**Fig. 11:**
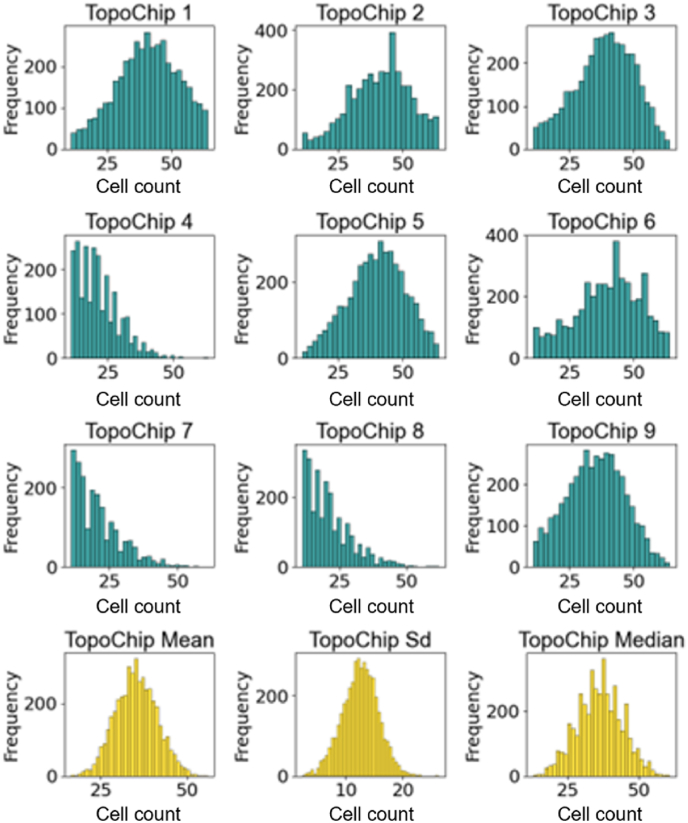
**Distribution of cell count on TopoChip surfaces**. TopoChip 1–9 (Blue): Individual histograms showing frequency distributions of cell counts of TopoUnits per TopoChip. TopoChip Mean, Sd, Median (Yellow)**:** Aggregate histograms representing the mean, standard deviation, and median of cell counts across the whole screen. (For interpretation of the references to color in this figure legend, the reader is referred to the Web version of this article.)

**Fig. 12:**
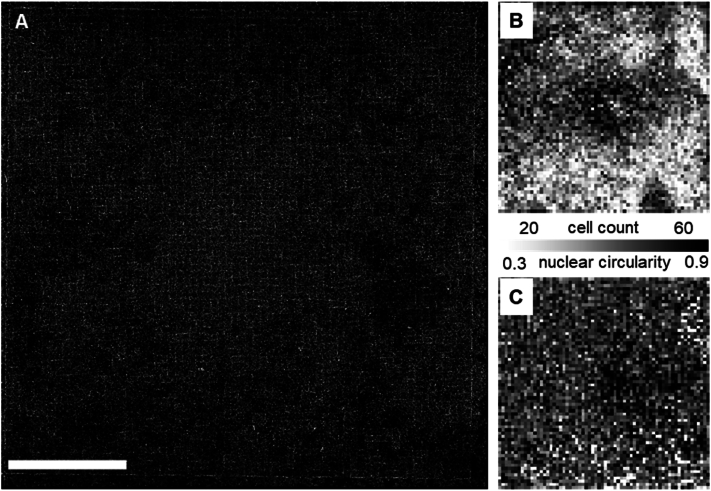
**Spatial variation in cell features across a TopoChip. (A)** Stitched image showing phalloidin signal of a whole TopoChip. **(B)** A heatmap displaying cell count per TopoUnit highlights significant regional variability. **The pseudocolor scale ranges from blue (low cell count) to yellow (high cell count). (C)** A heatmap displaying mean nuclear form factor per TopoUnit, calculated as (4×π×Area)/(Perimeter2). A value of 1 indicates a perfect circle, while lower values reflect more elongated or irregular shapes per TopoUnit. **The pseudocolor scale ranges from purple (low circularity) to yellow (high circularity).** Scale bar represents 500 μm.

Having established a dataset from which we removed artifacts at the TopoChip, object, and TopoUnit level, we next set out to explore the structure of the data and investigate the diversity of cellular features within it. Out of an initial 10 TopoChips prepared, data from 9 were successfully retrieved and included in the analysis following software-related issues with one chip. First, we investigated whether differences could be observed between these 9 TopoChips. To this end, we calculated the mean number of cells per TopoUnit for each of the TopoChips (Fig. 11). Interestingly, we observed that 6 out of these 9 TopoChips had 39 ± 13 cells per unit, whereas 3 TopoChips (chips 4, 7, and 8) had 15 ± 11 cells per unit. To assess if this difference originated from cell seeding or removal of outliers, we compared the DAPI signal of replicate TopoUnits across high and low cell count TopoChips. Visual inspection confirmed lower cell numbers on the three chips with low cell counts. Given the stochastic seeding method employed, and acknowledging the observed differences in cell density (as visually confirmed by DAPI signal comparison), we decided to retain data from all 9 TopoChips in the database, electing not to explicitly treat cell density as a confounding factor in the current analyses. However, for future exploration of this data set, the average seeding density could be considered as an exclusion criterion, or a feature to base new hypotheses on. As an example of such a hypothesis, we visually analyzed the relationship between the number of cells in a TopoUnit and some of the cellular features. In the stitched image of TopoChips (Fig. 12A), we observed regions of high and low phalloidin intensity across TopoChip 9. The mean number of cells per TopoUnit in this screen was 31 ± 12, and 90 % of the TopoUnits had a cell count between 25 and 39 cells. The color-coded map represents the heterogeneity of cell density which is caused by our cell seeding procedure. Next, we produced a similar map of TopoChip 9, but this time we plotted the mean nuclear circularity per TopoUnit, because we hypothesize that nuclear circularity depends on topography design, but not seeding density. Indeed, no regions of high or low circularity are observed on the chip.

**Fig. 13:**
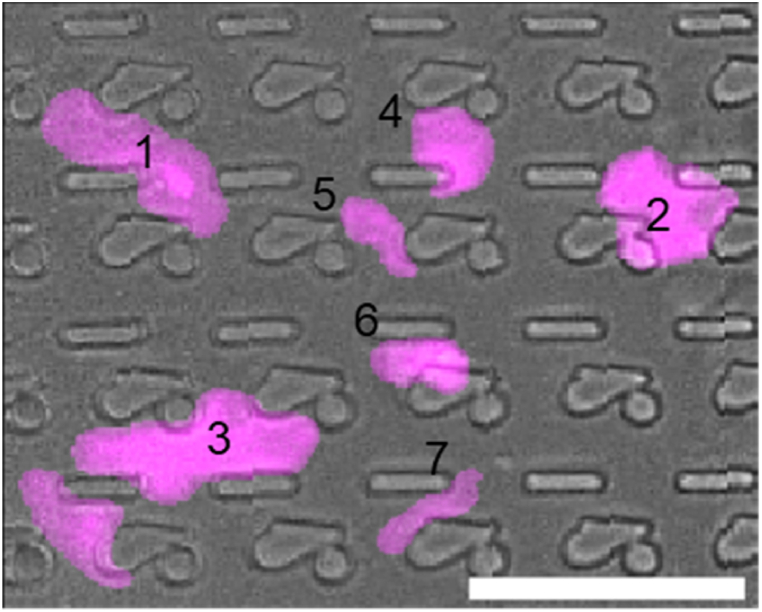
**Composite image demonstrating effect of topographical confinement on nuclear morphology.** Projections of fluorescently labeled podocyte nuclei (pink) onto corresponding design locations display variations in size and shape depending on their relative position to the underlying topographical features. Nuclei in areas of higher confinement (positions 4, 5, 6, 7) exhibit noticeably smaller and more constrained nuclei compared to those in wider confinement regions (positions 1, 2, 3). Scale bar represents 50 μm. (For interpretation of the references to color in this figure legend, the reader is referred to the Web version of this article.)

**Fig. 14:**
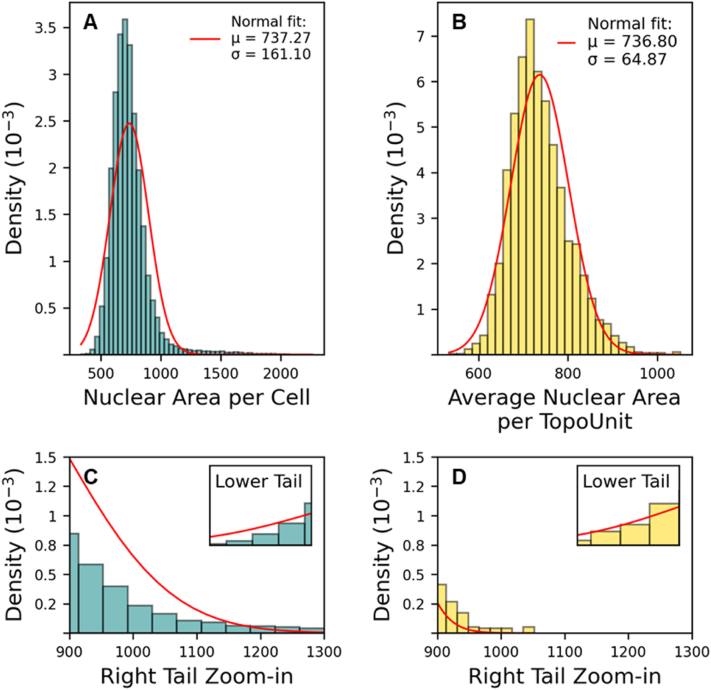
**Distribution of nuclear area at single-cell and TopoUnit level. (A)** Histogram showing the distribution of nuclear area measured at the single-cell level. The red line represents a fitted normal distribution (μ = 737, σ = 161). **(B)** Histogram of the average nuclear area per TopoUnit, where each bar reflects the mean of multiple single-cell measurements within one topography unit. A normal distribution fit is also shown in red (μ = 736, σ = 65). **(C, D)** Zoomed-in views of left and right tails (lower and higher nuclear area range) of the distributions in panels A and B, respectively. The y-axis shows probability density (10^−3^) in which the height of a bar reflects the relative probability of observing a nuclear area value within that bin. Both histograms are normalized so that the total area under the curve equals 1, enabling direct comparison of distribution shapes. (For interpretation of the references to color in this figure legend, the reader is referred to the Web version of this article.)

A second aspect we investigated was the variability in cell features within a TopoUnit. As we have seen in our earlier research, there is a strong correlation between TDDs and cell features such as cell area, nuclear shape, and expression of marker proteins [2,3,5]. However, we also observe substantial differences in the shape and size of cells and nuclei *within* a TopoUnit, clearly influenced by the exact location of the cell or nucleus relative to the pillars (Fig. 13). While our current machine learning models do not incorporate the precise location of individual cells, acknowledging this micro-variability could potentially enhance predictive power. To further investigate the distribution of nuclear area, we plotted histograms for nuclear area at the single-cell level and for the mean nuclear area per TopoUnit (Fig. 14). The mean nuclear area per TopoUnit followed a Gaussian distribution with an average of 737 and a standard deviation of 65 (Fig. 14B). At the single-cell level, the average nuclear area was similar (737), but the standard deviation was considerably higher (161) (Fig. 14A). This increased variability at the single-cell level reflects the broad range of individual cell responses to local topographical features, whereas the narrower distribution of mean nuclear area per TopoUnit is a statistical consequence of averaging multiple measurements within each unit. The histogram plot of individual cell nuclear area was right-skewed, featuring a subset of cells with considerably larger nuclei.

### 3.7 Surface ranking and machine learning analysis

**Fig. 15:**
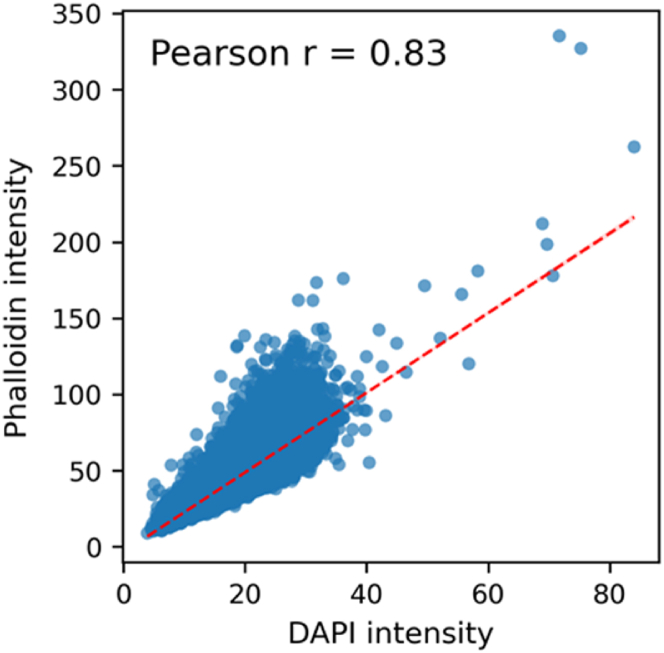
**Correlation between DAPI and phalloidin intensities within DAPI-segmented regions.** A scatter plot showing the relationship between mean DAPI intensity and mean phalloidin intensity measured within DAPI-defined nuclear regions. Each point represents a single segmented nucleus. A strong positive correlation was observed (Pearson *r* = 0.83). A linear regression trendline is shown in dashed red. (For interpretation of the references to color in this figure legend, the reader is referred to the Web version of this article.)

To illustrate the depth and versatility of the dataset, we conducted a series of exploratory analyses across more than 1.1 million segmented cells and 31,000 TopoUnits. The analyses were not intended to uncover new biological mechanisms, but rather to demonstrate the structural integrity, statistical richness, and reusability of the dataset. We first examined correlations between nuclear and cytoskeletal signals. We and others have shown that nuclear confinement results in higher DAPI signal due to epigenetic remodelling of the chromosomes [38,39,48]. We hypothesize that cells will also increase peri-nuclear F-actin to provide mechanical protection. To this end, we analyzed DAPI and phalloidin integrated intensity in the nuclear region. We indeed observed a strong positive correlation between mean DAPI intensity and mean phalloidin intensity (Pearson r = 0.83), suggesting that nuclei with more compact DNA also exhibit enriched actin signal near or within the nuclear periphery (Fig. 15).

**Fig. 16:**
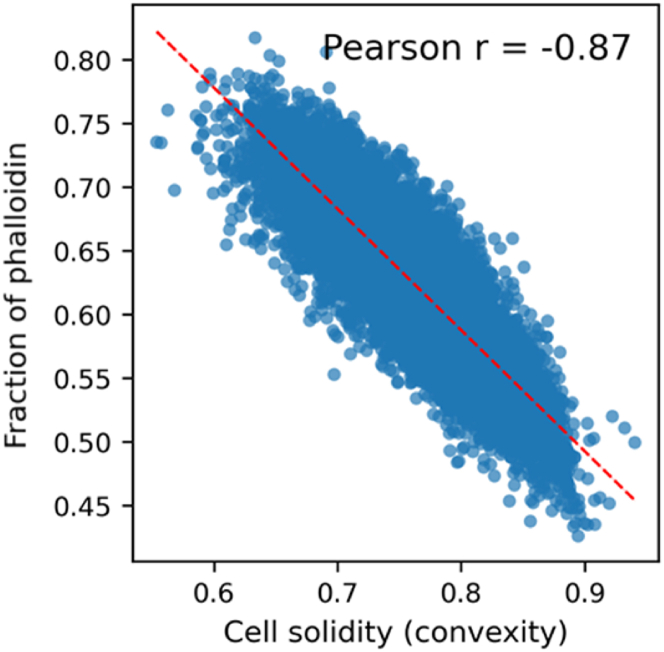
**Correlation between cell solidity and peripheral phalloidin localization.** Scatter plot showing the relationship between cell solidity (a measure of convexity) and the fraction of phalloidin signal localized in the outermost radial region of the cell (the 4/4 bin). Each point represents a single segmented cell. A strong negative correlation was observed (Pearson r = −0.87), suggesting that less convex (more irregular) cells exhibit greater peripheral accumulation of actin. A linear regression trendline is shown in dashed red. (For interpretation of the references to color in this figure legend, the reader is referred to the Web version of this article.)

Next, we assessed how cell shape relates to the localization of F-actin. We noticed that some podocytes on the TopoChip display intense cortical actin and to investigate a relationship between podocyte shape and F-actin localization, we compared cell solidity (a feature describing boundary convexity) with the fraction of phalloidin signal in the outermost radial bin of the cell. Here, a strong negative correlation emerged between cell solidity and the fraction of phalloidin signal localized to the outermost radial bin of the cell (Pearson r = −0.87). Cells with more irregular, protrusive outlines tended to accumulate actin at their peripheries, while smoother, more convex cells showed more centralized actin distribution (Fig. 16). This finding confirms that the dataset preserves biologically interpretable relationships between cell morphology and subcellular architecture. In further analyses we discovered that cell solidity positively correlated with cell form factor (Pearson r = 0.92) and negatively with cell compactness (Pearson r = −0.91), while DAPI intensity correlated strongly with both total and edge-localized nuclear signal features (Pearson r > 0.93). Data are available at https://github.com/cbite/ciPODs_9_PS_TopoChips_SCREEN_NK_MGV_14022025. These relationships demonstrate that our segmentation and feature extraction pipelines yielded quantitatively robust and biologically plausible outputs.

### 3.8 LightGBM-based classification to illustrate predictive utility of topography features

**Fig. 17:**
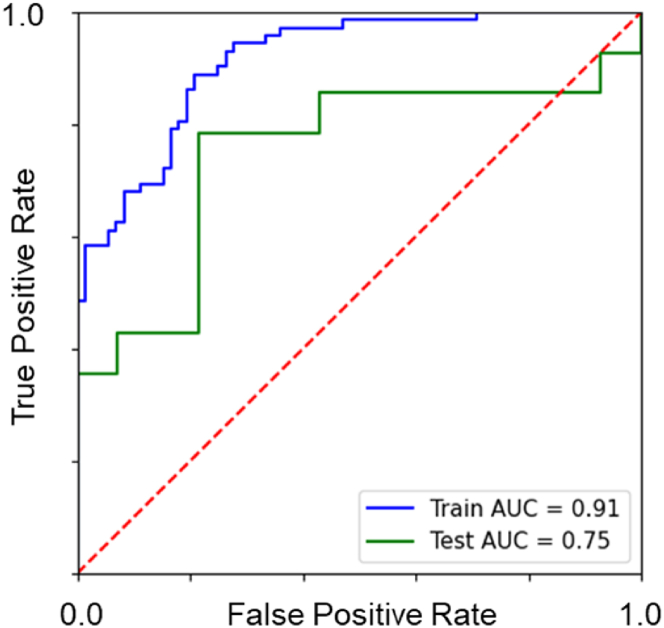
**Cell count classification model.** Receiver operating characteristic (ROC) curves of the final LightGBM classification model for both the training set (blue) and the test set (green). The dashed red diagonal denotes the random‐guess baseline. (For interpretation of the references to color in this figure legend, the reader is referred to the Web version of this article.)

**Fig. 18:**
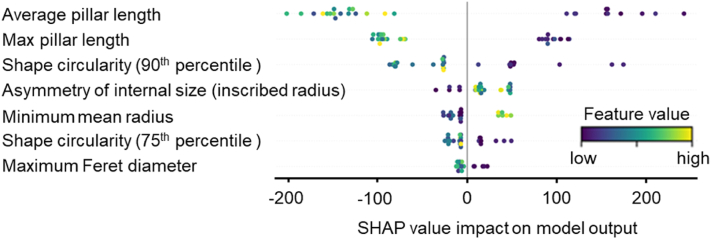
**Features that model the relation between topography design descriptors and cell count.** Each row corresponds to one feature (sorted by overall importance), and each dot represents a single sample (TopoUnit). The position on the horizontal axis reflects the feature's contribution (SHAP value) to the model output, with positive values pushing the prediction toward the “positive” class and negative values pushing it toward the “negative” class. The color scale (blue or yellow) indicates the actual feature value, with blue representing lower feature values and red higher. (For interpretation of the references to color in this figure legend, the reader is referred to the Web version of this article.)

To demonstrate the predictive potential of our dataset and the reusability of its annotations, we trained a LightGBM classifier to classify high- and low-performing surface topographies based on podocyte cell count. We selected the 90 top and 90 bottom-ranked surfaces and used TDDs as input features. We structured the experiment as a binary classification task and split the data into training, validation, and test subsets (with stratification). To mitigate overfitting and assess repeatability, the training procedure was replicated twice, and models were optimized using Optuna's Tree-structured Parzen Estimator (TPE) sampler with a multi-objective optimization strategy. The final model demonstrated robust classification performance, achieving a high ROC-AUC score on both training and validation datasets (Fig. 17). Notably, the model maintained strong balanced accuracy, indicating resilience to potential class imbalance. Full model configuration, parameters, and performance metrics are accessible in our GitHub repository, providing a transparent and reproducible benchmark for future research. To enhance interpretability of the model, we used SHAP (SHapley Additive exPlanations) to quantify the contribution of each feature on classification outcome. The resulting SHAP summary plot (Fig. 18) revealed biologically intuitive patterns—for example, topography features such as pillar length, shape circularity, internal asymmetry, and surface roughness were among the most predictive. These insights confirmed that the model effectively identified which design attributes were most influential in shaping cellular behavior, and reflected the biological relevance of topographic modulation. We emphasize that this machine learning exercise is intended as a proof-of-capability rather than an exhaustive exploration. The database is intentionally modular and designed to support a broad spectrum of ML tasks—ranging from multi-class classification and regression to unsupervised clustering, feature selection, or even few-shot transfer learning. Researchers are encouraged to reuse the annotated data, segmentation outputs, and codebase to explore alternative questions, build benchmarks, or develop new methods in computational cell biology and biomaterials design.

## 4 Discussion

The FAIR principles—Findable, Accessible, Interoperable, and Reusable—provide a robust framework for sustainable data stewardship. In this study, we aligned a high-throughput podocyte imaging workflow developed on the TopoChip platform with these principles, aiming to make our datasets more transparent, reproducible, and reusable [29,36]. Findability was enhanced by systematically organizing all datasets and embedding rich metadata. The raw image dataset—over 130,000 files across three fluorescence channels—is hosted locally and available via request, with filenames encoding experimental variables such as TopoUnit coordinates, stain identity, magnification, and chip ID. This structured convention allows automated parsing and ensures traceability. Since no dedicated repository exists for image-based biomaterials data (unlike GEO for transcriptomics), we complemented public hosting with archiving on institutional servers (TU/e), addressing long-term accessibility and provenance [22]. Accessibility was addressed by releasing not only raw images but also a curated dataset of 1.2 million segmented cells and 6.6 billion extracted features. These are supported by open-access pipelines (e.g., CellProfiler) [49], custom scripts (topocropper.m, removeOutliers.py), and SOPs—all version-controlled to ensure reproducibility. While prior efforts have demonstrated the feasibility of high-content image analysis [25,30], they often lacked documentation of underlying assumptions. Our workflow includes additional metadata (e.g., z-stack projection parameters), though some variables concerning the experimental protocol remain unstructured—highlighting ongoing challenges in experimental metadata capture. Interoperability was promoted through use of biomedical ontologies including OBI (Ontology for Biomedical Investigations), CL (Cell Ontology), and EDAM. These enabled semantic annotation of experiments and outputs, allowing easier integration with external resources [37,50]. Our approach aligns with standards like MIACME, the JUMP Cell Painting dataset and Nottingham Research Data Management Repository by integrating ontological mapping and structured metadata layers. We aimed to promote transparency and reusability by explicitly documenting the rationale behind key image processing choices—such as z-stack projection methods and parameter selection—thereby supporting better downstream integration and interpretation [24]. Reusability is supported through a multi-tiered quality control framework spanning the TopoChip, TopoUnit, and object levels. This included biologically relevant thresholds (e.g., expected cell counts, nucleus-to-cell area ratios) paired with adaptive filtering techniques such as IQR-based exclusion and Anderson-Darling tests. Several published datasets, including our own, emphasize segmentation-based profiling but often lack clear exclusion criteria [5,49]. Our contribution includes reusable QC scripts and proposed internal controls (e.g., landmark channels or universal stains), analogous to housekeeping genes in transcriptomics [22].

While image acquisition is essential for spatial fidelity, our experience indicates it is not the primary throughput bottleneck. Scanning a full TopoChip takes approximately 6 h at 20 × magnification; however, segmentation tuning, data curation, and quality control require more time, expertise, and computational resources. CellProfiler pipeline optimization, manual segmentation review, and scripting-based outlier removal were particularly resource-intensive. To improve efficiency, future pipelines may benefit more from automated QC and machine learning–driven segmentation assessment than from faster acquisition hardware alone [51]. Semi-supervised learning models trained on manually verified segmentations can detect under- or over-segmentation [52] and flag low-confidence objects [53]. These models reduce reliance on expert oversight while preserving biological nuance. Similar approaches in digital pathology and single-cell imaging have improved consistency and reduced annotation fatigue [54]. Incorporating active learning strategies—where models prioritize uncertain or informative cases for human annotation—could further optimize curation efforts [55]. We anticipate such AI-augmented QC systems will be essential for maintaining dataset fidelity and reproducibility at scale. To accelerate acquisition while preserving segmentation quality, we envision integrating super resolution generative modelling (e.g. SRGANs) [56]. These models reconstruct high-resolution images from lower-magnification inputs by learning spatial representations. As SRGANs improve in fidelity, they could allow imaging at 10 × rather than 20 × magnification—cutting scanning time per chip substantially without losing analytical value [53]. SRGANs could also enable Dataset Condensation (DC)—a method to extract representative subsets from massive datasets. We previously clustered cells based on morphology and selected 28 prototypical shapes to span the observed variance [57]. DC would extend this approach by algorithmically identifying a minimal set of TopoUnits that capture the full morphological landscape [2,5,58,59]. This would reduce training set redundancy, ease manual validation, and support more efficient hypothesis testing.

We implemented a three-layered quality control (QC) framework across the TopoChip, TopoUnit, and object levels. Filters included cell count thresholds, nucleus-to-cell ratios, and eccentricity measures, followed by manual expert review. This design removed artifacts and biologically implausible segmentations from the final dataset [4]. To further increase robustness, we plan to incorporate models trained on verified segmentations, combining automation with human expertise [51]. Segmentation quality was validated through visual inspection of over 1000 TopoUnits by an imaging expert and two podocyte specialists. This qualitative strategy ensured plausibility but lacked quantitative inter-observer agreement or a ground truth benchmark [3]. Future workflows should incorporate curated annotations and consensus scoring to support formal benchmarking—particularly for morphologically complex cells like podocytes [37]. Our cell-count thresholds (11–64 cells per TopoUnit) were informed by an empirical analysis of feature variability across TopoChips. As shown in Fig. 9, the coefficient of variation for multiple morphological descriptors increased markedly when counts fell below 11 or exceeded 64, reflecting sampling noise at low density and multilayering at high density. Although this inevitably excludes some potential true phenotypic responses, the elevated variance in these regimes prevents robust comparisons. We therefore defined 11–64 cells as a stability band that supports reliable interpretation, while recognizing that out-of-band TopoUnits may still be of exploratory interest and could be revisited in targeted follow-up assays. Although this study focused on podocytes as a morphologically rich and clinically relevant model, the workflow was designed to be cell type-agnostic. Segmentation pipelines can be adapted by substituting lineage-specific markers (e.g., actin in MSCs, CD45 in immune cells), and QC thresholds can be tuned to reflect differences in morphology or cell density. The broader FAIR and metadata framework is independent of the chosen biology and therefore directly transferable to other TopoChip screens. We note, however, that applying the workflow to alternative materials such as polymer libraries may introduce unique challenges, including autofluorescence from certain chemistries or uneven staining due to surface chemistry effects. These artifacts can be mitigated through strategies such as spectral unmixing, careful marker selection, or background subtraction, but their impact should be explicitly benchmarked in future work. Establishing annotated, FAIR-compliant datasets across multiple cell types and material platforms will be an important step toward demonstrating interoperability and ensuring uptake by the wider biomaterials community. A further limitation is that reproducibility was only established within a single laboratory, and cross-laboratory benchmarking across different microscopes, operators, and staining protocols remains an essential future step. The LightGBM classifier trained on extracted features demonstrated high accuracy, interpretability via SHAP values, and strong ROC-AUC scores. However, it was trained on only 180 of 1835 surfaces, raising concerns around overfitting and generalizability [29]. While effective for podocyte responses, the model was not evaluated across cell types or dynamic conditions. It also lacked omics integration, limiting its ability to infer underlying biological mechanisms [2]. While we draw analogies to transcriptomics in terms of scale and metadata structuring, we recognize that omics fields advanced through the establishment of community standards and mechanistic integration. Our current dataset is tightly coupled to the TopoChip platform, but many aspects of the pipeline are platform-agnostic, including image acquisition logic, segmentation, QC filtering (cell count stability, replicate consistency, NtoC ratio), and FAIR metadata structuring. Elements that are TopoChip-specific include the coordinate-based file naming convention and geometric descriptors tied to the micro-patterned topographies. Adapting this framework to other high-throughput materials platforms (e.g., polymer microarrays or chemistry-based combinatorial libraries) would primarily require adjusting the metadata schema to reflect material identity and modifying topography descriptors. Importantly, the layered QC logic, ontological annotation, and dataset archiving approach are directly transferable, and thus we view this work as a foundation for cross-platform image data standardization rather than a siloed dataset. While the present study focuses on morphological profiling, functional validation represents a critical future step. For example, nephrin clustering is a hallmark of podocyte health and could be correlated with the morphological descriptors reported here. Likewise, integration with transcriptomic or proteomic profiling would allow a multi-omics approach linking topography-induced morphological changes to underlying molecular pathways. Such strategies would not only extend biological interpretability but also bridge this dataset to domains where large-scale repositories and community standards already exist, thereby facilitating broader adoption and interoperability [60,61]. Future models should include replicates in training/test splits, be validated across multiple screens, and incorporate molecular or time-lapse data to enhance mechanistic insight [50]. This will improve predictive power and robustness for downstream material design applications. With 660 GB of raw images and gigabyte-scale segmentation outputs, sustainability and scalability are major concerns [62]. Human validation was a critical bottleneck—especially in the absence of dedicated podocyte or ML experts [63]. To address this, we implemented FAIR-aligned archiving with metadata compression, ontological annotation, and transparent sharing of model training scripts and QC parameters. However, as dataset size grows, ensuring clear documentation of assumptions will be critical to avoid user misinterpretation [62,64]. Continued emphasis on dataset condensation, distributed computing, and automation will be essential for sustainable, ethical reuse [65]. Despite growing public access to large-scale imaging datasets, the ability to effectively analyze such data remains largely restricted to technically equipped labs. Major barriers include the learning curve of tools like CellProfiler or Python-based pipelines, lack of standardized formats, and the computational demands of working with terabyte-scale data [66]. Democratization, in this context, means lowering the barriers for researchers of all backgrounds to access, analyze, and draw insights from imaging data. This goal intersects with education, infrastructure, and AI in meaningful ways. Educationally, image informatics [[67], [68], [69]] and FAIR data handling should be integrated into standard life sciences training. Basic skills like segmentation logic, dimensionality reduction, and model interpretation should accompany wet-lab protocols. In terms of infrastructure, future repositories should support interactive querying and visualization, allowing users to explore segmented features or annotate datasets through web-based interfaces. Projects like the Allen Cell Collection [27] and the Cell Painting Gallery [26] portal illustrate this shift. Perhaps most transformative is the role of AI-powered interfaces. Low-code platforms and foundation models make it feasible to build natural language interfaces that allow users to ask questions like, “Which topographies increase nuclear eccentricity under condition X?”—without programming knowledge [70]. Combined with containerized dashboards (e.g., using JupyterLite or Streamlit), these tools could radically broaden accessibility. Ultimately, the goal is to ensure that the scientific value of large-scale imaging data is not confined to a few expert groups but becomes a shared, reusable asset across the community.

## 5 Conclusion

In this work, we introduce a scalable, reproducible, and FAIR-aligned framework for large-scale imaging and analysis of podocyte responses to micro-structured biomaterial surfaces. By integrating high-resolution imaging, standardized segmentation, rigorous quality control, and open data infrastructure, we contribute one of the most comprehensive reference datasets for in silico cell–biomaterial research to date. Beyond demonstrating feasibility, our pipeline offers a modular architecture that others can adapt, reuse, and extend across cell types, imaging platforms, and screening modalities. The dataset, scripts, and annotations are made openly available to support machine learning benchmarking, phenotypic discovery, and educational use. This work lays the foundation for the next generation of image-based biomaterial screening—one that is transparent, collaborative, and driven by community standards. Future efforts will focus on expanding the dataset's dimensionality (e.g., 3D segmentation, transcriptomics integration), enabling federated data pooling, and developing user-friendly interfaces for non-experts, thus advancing truly democratized in silico experimentation.

## CRediT authorship contribution statement

**Nikita Konshin:** Writing – review & editing, Writing – original draft, Visualization, Software, Methodology, Investigation, Formal analysis, Data curation, Conceptualization. **Marta Garcia Valverde:** Writing – review & editing, Data curation. **Danila Solodennikov:** Methodology, Formal analysis. **Koen Minartz:** Writing – review & editing, Software. **Vlado Menkovski:** Writing – review & editing. **Rosalinde Masereeuw:** Writing – review & editing. **Shantanu Singh:** Writing – review & editing. **Silvia M. Mihăilă:** Writing – review & editing. **Jan de Boer:** Writing – review & editing, Writing – original draft, Supervision, Resources, Project administration, Methodology, Investigation, Funding acquisition, Conceptualization.

## Declaration of generative AI and AI-assisted technologies in the writing process

During the preparation of this work, the authors used **ChatGPT (OpenAI)** for language refinement, manuscript structure optimization, and guidance on compliance with journal submission guidelines. After using this tool, the authors carefully reviewed and edited the content as needed and take full responsibility for the accuracy, completeness, and integrity of the published article.

## Declaration of competing interest

The authors declare the following financial interests/personal relationships which may be considered as potential competing interests:Nikita Konshin reports financial support was provided by 10.13039/501100003005Eindhoven University of Technology. If there are other authors, they declare that they have no known competing financial interests or personal relationships that could have appeared to influence the work reported in this paper.

## Data Availability

Data will be made available on request.
